# Milk‐derived exosomes in the regulation of nutritional and immune functions

**DOI:** 10.1002/fsn3.4323

**Published:** 2024-08-21

**Authors:** Hui Yang, Tana Wuren, Bin‐tao Zhai, Yang Liu, Demtu Er

**Affiliations:** ^1^ College of Basic Medical Science Qinghai University Xining Qinghai PR China; ^2^ Research Center for High Altitude Medicine Qinghai University Xining Qinghai PR China; ^3^ Key Laboratory of Veterinary Pharmaceutical Development, Lanzhou Institute of Husbandry and Pharmaceutical Sciences Chinese Academy of Agricultural Sciences Lanzhou Gansu PR China; ^4^ College of Life Science Ningxia University Yinchuan Ningxia PR China; ^5^ College of Veterinary Medicine Inner Mongolia Agricultural University Hohhot Inner Mongolia PR China

**Keywords:** exosomes, immunity, milk, nutrition, signaling molecules

## Abstract

Milk‐derived exosomes (MDEs), being a component of milk, have the potential to support immune system maturation in offspring and enhance immune cell proliferation. Through the transport and transmission of essential signaling molecules, MDEs contribute to the regulation of intergenerational and intraspecies communication, thereby impacting nutrient uptake and metabolic functions. A comprehensive comprehension of MDE functionalities is imperative for enhancing the quality of the dairy industry. A systematic search of the databases PubMed/Medline, Web of Science, and Scopus utilizing predetermined keywords resulted in the identification of 418 articles, of which 67 were chosen for inclusion in this review, which specifically explores the intersection of immune response and nutrition. This article provides a critical analysis of the classification of extracellular vesicles, the mechanisms underlying the biosynthesis of microvesicular dietary exosomes (MDEs), the components of MDEs, and their relevance in the contexts of nutrition and immune modulation. The primary aim of this review was to offer valuable scholarly insights to support the advancement and practical application of MDEs.

## Graphical abstract

Milk‐derived exosomes (MDEs), which are diminutive vesicular entities harboring diverse bioactive constituents, have been demonstrated to exert a significant influence on nutrition and immune function regulation. The constituents of MDEs possess the ability to suppress inflammatory responses, fortify the integrity of the intestinal barrier, mitigate intestinal harm, and exhibit promising prospects for the prevention and treatment of intestinal disorders. Furthermore, they may augment immune functionality through the modulation of immune cell behavior and intercellular communication. Collectively, MDEs represent biologically active agents with the potential to regulate nutrition and immune responses, thereby offering a means to enhance human well‐being and avert ailments.

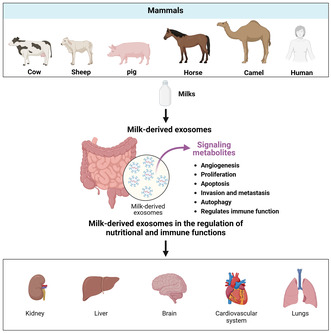

## 1 INTRODUCTION

Mammalian breast milk is a complex mixture of nutrients, including proteins, lactose, fat, vitamins, minerals, and biologically active substances such as neuronal growth factors, growth regulatory factors, and immune‐related factors (Atyeo & Alter, 2021; Zhou & Pu, 2023). This intricate composition is essential for the growth and development of infants and the maturation of their immune system. Milk‐derived exosomes (MDEs), produced by mammary epithelial cells, play a role in transporting bioactive molecules (Cui et al., 2023; Lu et al., 2024). The principal components of key signaling molecules carried by MDEs include lipids, proteins, and nucleic acids (e.g., mRNA, miRNAs, lncRNA, and DNA) (Martínez‐Santillán & González‐Valdez, 2023; Peng et al., 2023; Potrich et al., 2024). These molecules are capable of being internalized by recipient cells to exert specific biological functions, such as modulating cell proliferation and regulating apoptosis (Cui et al., 2023; Lu et al., 2024; Martínez‐Santillán & González‐Valdez, 2023). A growing body of research indicates that microRNAs present in extracellular vesicles derived from milk are involved in various physiological processes, including immune cell maturation, modulation of immune responses, formation of neuronal synapses, and the development of metabolic disorders such as obesity and diabetes (Ahmed et al., 2022; Gong et al., 2023; Han et al., 2024). This underscores the potential efficacy of milk‐derived extracellular vesicles in functional foods (Ahmed et al., 2022; Han et al., 2024).

Several research studies have shown that milk from various mammalian species, including humans, cows, pigs, pandas, sheep, rats, and wallabies, contains a substantial amount of exosomes (Zeng, Chen, et al., 2021). These exosomes, enclosed by a lipid membrane, safeguard milk‐derived RNAs from degradation by RNases, acidic conditions, and digestive enzymes, thus preserving their crucial functions in intercellular communication between mothers and offspring (Ahmed et al., 2022; Han et al., 2024; Zeng, Chen, et al., 2021). In vitro studies have demonstrated that RNA molecules originating from milk exosomes have the ability to be internalized by intestinal and immune cells (Cui et al., 2023; Han et al., 2024). Following the introduction of labeled bovine milk exosomes, it was observed that these exosomes, along with their RNA payload, were able to enter the circulatory system and disseminate to multiple tissues in mice (Han et al., 2024; Zeng, Chen, et al., 2021). Moreover, it has been observed that miRNAs present in milk exosomes demonstrate notable sequence similarities, particularly within the let‐7 family (let‐7a, let‐7b, and let‐7f) and miR‐148a, which exhibit conserved sequences across diverse species (Cui et al., 2023; Han et al., 2024; Martínez‐Santillán & González‐Valdez, 2023; Potrich et al., 2024). Additionally, in conjunction with their shared sequence homology, milk exosomes also demonstrate interspecies tolerance (Zeng, Chen, et al., 2021). The transfer of highly expressed let‐7 family and miR‐148a from milk exosomes to neonatal organisms may contribute to posttranscriptional regulation of target mRNA, thereby facilitating interspecies communication (Cui et al., 2023; Zeng, Chen, et al., 2021). Therefore, exploring the biological mechanisms of MDEs could have substantial implications for both basic scientific research and clinical applications.

In light of the growing interest in MDEs and their potential impact on human health, the aim of this study was to comprehensively examine their classification, synthesis, absorption mechanisms, nutritional regulation, and immunological significance. Through this investigation, we seek to offer valuable insights that will enhance our comprehension of MDEs biology and serve as essential reference materials for future research endeavors focused on harnessing the therapeutic properties of MDEs.

## 2 METHODS

A comprehensive literature review was conducted using three prominent electronic databases, namely PubMed/Medline, Web of Science, and Scopus, with predefined keywords until March 2024. The focus of the search was on the topic of “Milk‐derived exosomes” in conjunction with the terms “immune” and “nutrition.” A comprehensive analysis was conducted on 418 articles, comprising 327 original research articles and 91 review articles. Additionally, the reference lists of the identified articles were scrutinized to ascertain additional pertinent literature. Ultimately, 75 articles centered on immune response and nutrition were selected for incorporation in this literature review.

## 3 CLASSIFICATION OF EXTRACELLULAR VESICLES

**FIGURE 1:**
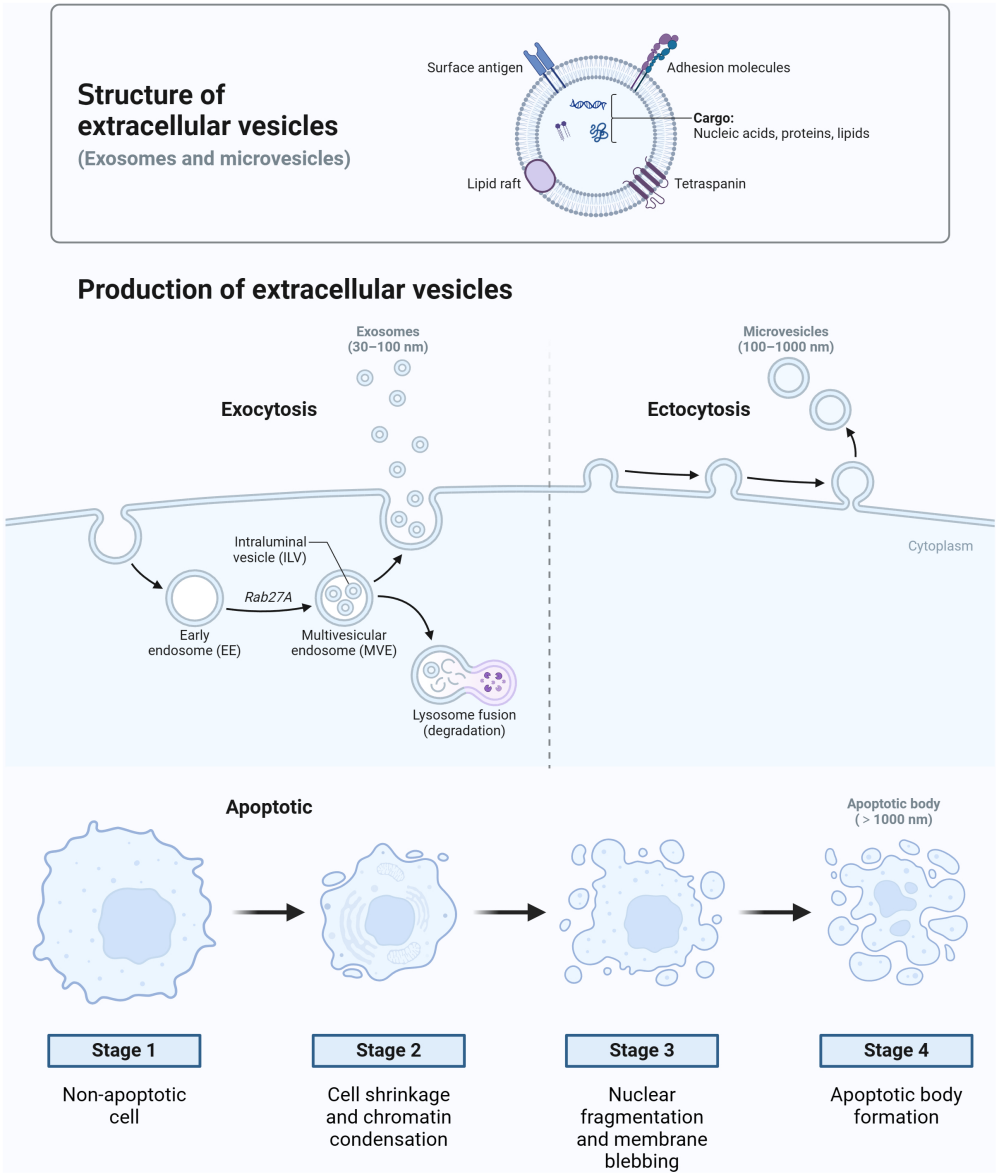
Biogenesis of three primary subtypes of extracellular vesicles: Exosomes, microvesicles, and apoptotic bodies.

Extracellular vesicles (EVs) are a class of functional and dynamic nanoscale membrane‐bound vesicles that are secreted by all cells (Cocozza et al., 2020; Gupta et al., 2021; Simpson, 2017). They are categorized into three distinct groups: exosomes, microvesicles, and apoptotic bodies, each possessing unique structural, origin, and biological characteristics (Figure 1) (Cable et al., 2023; Gupta et al., 2021; Wen et al., 2023). Specifically, exosomes and microvesicles differ in their origin and release mechanisms, with exosomes being primarily secreted by cells and their production and secretion being dependent on the state and function of secretory cells (Cable et al., 2023; Gupta et al., 2021; Wen et al., 2023). In contrast, microvesicles can be generated by any active cell type and exhibit a more ubiquitous and stochastic release pattern (Cable et al., 2023; Gupta et al., 2021; Simpson, 2017). Additionally, the primary distinction between exosomes and apoptotic bodies pertains to how they are produced (Cable et al., 2023). While apoptotic bodies arise from cellular self‐dissolution during apoptosis, exosomes and microvesicles are spontaneously released rather than actively secreted (Cable et al., 2023). Furthermore, the three distinct categories of exosomes exhibit differences in their respective targets, transported substances, and physiological impacts (Cable et al., 2023; Cocozza et al., 2020; Gupta et al., 2021). Exosomes are known to transport informational molecules, such as RNA, proteins, and other biological entities, including viruses and other small molecules (Cable et al., 2023; Cocozza et al., 2020). Microvesicles, however, carry a diverse array of biological molecules, including membrane proteins, peptides, and metabolites (Cable et al., 2023; Simpson, 2017; Wen et al., 2023). Finally, apoptotic bodies have the capacity to release cytokines, which can induce inflammation and attract immune cells to specific sites (Kholodenko et al., 2022; Santavanond et al., 2021; Zhou et al., 2022). In summary, exosomes, microvesicles, and apoptotic bodies exhibit notable differences in their structural composition, physiological processes, and functional properties (Table 1) (Cable et al., 2023; Simpson, 2017; Wen et al., 2023). In both clinical and fundamental research, precise differentiation of these entities can facilitate a more comprehensive understanding of their respective roles and mechanisms, thereby offering novel insights and approaches for the prevention and treatment of associated pathologies.

**TABLE 1:** Classification of extracellular vesicles.

Classification	Exosomes	Microvesicles	Apoptosis bodies
Diameter (nm)	40–150 nm	150–1000 nm	>1000 nm
Density (g/mL)	1.11–1.19 g/mL	1.02–1.22 g/mL	1.16–1.28 g/mL
Source	Intracellular vesicular vesicles fuse with the cell membrane and are released outside the cell.	Cell membrane budding formation.	Released after cell apoptosis.
Protein markers	Alix, TSG101, HSC70, CD63, CD81, and CD9	Selectin, integrins, CD40, and MMP	Histones
Carrying substances	mRNA, miRNA, and DNA	mRNA, miRNA, and DNA	mRNA, miRNA, and DNA
Function	Intracellular information transmission and extracellular material exchange.	Intracellular information transmission and intercellular genetic material transport.	Regulating pathological and physiological processes.

## 4 BIOGENESIS OF EXOSOMES

**FIGURE 2:**
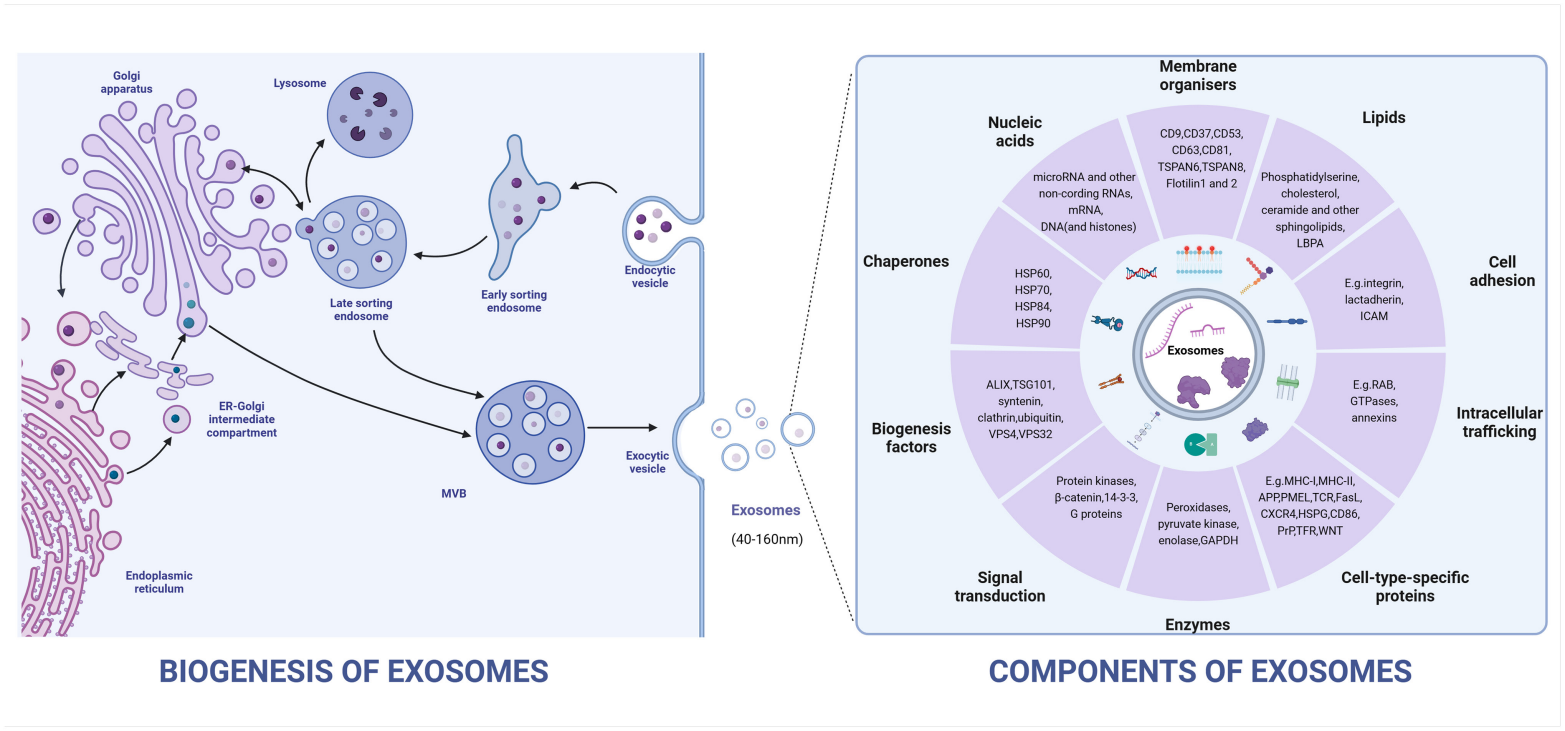
Formation and components of exosomes: Generated via cell membrane endocytosis, including proteins (tetraspanins, annexin, heat shock proteins), lipids (ceramide, cholesterol, phosphatidylserine), and nucleic acids (DNA and RNA).

The generation of MDEs is governed by a complex series of biological processes that involve packaging of proteins and RNA within the cell into small vesicles connected to the plasma membrane via endosomes (Figure 2) (Cocozza et al., 2020; Martínez‐Santillán & González‐Valdez, 2023; Zeng, Chen, et al., 2021). This process is primarily regulated by the endoplasmic reticulum and influenced by the cytoskeleton, which includes microfilaments and microtubules (Cable et al., 2023; Zeng, Chen, et al., 2021). MDEs are formed through the budding of intraluminal vesicles (ILVs) from the nuclear membrane during the maturation of multivesicular endosomes (MVEs) (Ahmed et al., 2022; Zeng, Chen, et al., 2021). Through the nuclear membrane system, certain MVEs undergo fusion with the cytoplasmic membrane, leading to the release of ILVs that constitute MDEs (Ahmed et al., 2022; Lönnerdal, 2019; Zeng, Chen, et al., 2021). In contrast, other MVEs are degraded by lysosomes (Lönnerdal, 2019). Exosome biogenesis requires the participation of the endosomal sorting complex required for transport (ESCRT), which comprises > 30 proteins and forms four distinct complexes (ESCRT‐0, I, II, and III), along with vacuolar protein sorting 4 (VPS4), vesicle trafficking 1 (VTA1), and programmed cell death 6 interacting protein (ALIX). The ESCRT‐0 complex facilitates the recruitment of sorting complexes to MVBs in a ubiquitination‐dependent manner, followed by the recruitment of ESCRT‐I to the nuclear membrane (Cui et al., 2023; Lönnerdal, 2019; Stefanon et al., 2023). ESCRT‐I recruits both ESCRT‐II and ESCRT‐III, which facilitate bud formation and vesicle release, respectively. VPS4 subsequently provides the necessary energy to disassemble ESCRT‐III from the intraluminal vesicle membrane for recycling (Ju et al., 2021; Kenific et al., 2021; Lee et al., 2023). Recent studies have revealed the existence of ESCRT‐independent mechanisms in exosome production, which are associated with the activities of lipids, four‐span transmembrane proteins, and heat shock proteins (Ju et al., 2021; Lee et al., 2023). For instance, the transport of extracellular vesicle‐related domains into the intraluminal cavity of the nuclear membrane does not rely on ESCRT function but instead requires sphingolipid ceramide (Ju et al., 2021; Kenific et al., 2021; Lee et al., 2023). Lysosomal‐associated membrane protein 3 (CD63) in human melanoma cells facilitates the sorting of melanosome proteins into ILVs, independent of ceramide or ESCRT, and its core participating proteins are shown in Table 2 (Ju et al., 2021; Kenific et al., 2021; Lee et al., 2023). Exosome transport involves the member RAS oncogene family (Rab) protein family of Guanosine Triphosphate (GTPases), Rab11, Rab7, Rab35, and member RAS oncogene family 11 (Rab27a), which play crucial roles in vesicle budding, fusion, and release (Cui et al., 2023; Han et al., 2024; Lee et al., 2023). Exosome secretion requires the involvement of soluble NSF attachment protein receptor (SNARE) proteins and the synaptic protein family (Han et al., 2024; Kenific et al., 2021; Zeng, Chen, et al., 2021). The advancement of mass spectrometry technology and omics methodologies has facilitated numerous investigations into the transcriptome, proteome, lipidome, and metabolome of exosomes, with the primary objective of accurately characterizing their composition (Lönnerdal, 2019; Potrich et al., 2024; Zeng, Chen, et al., 2021). These studies have established a robust research framework for exploring the biogenesis, release, and cargo sorting mechanisms of exosomes. The complex mechanism still requires further comprehensive research.

**TABLE 2:** Research on the biogenesis and release of exosomes.

Description	Protein	Used for exosome definition	References
ESCRT‐dependent	HRS	MHC‐II, VPS4B, Tsg101, CD63, HSC70, CD81	Ju et al. (2021), Lee et al. (2023)
	STAM1	CD63, CD81, MHC‐II, HSC70	Cui et al. (2023), Lönnerdal (2019), Stefanon et al. (2023)
	Tsg101 (VPS23)	CD63, CD81, MHC‐II, HSC70, syndecan‐1, ALIX	Ju et al. (2021), Kenific et al. (2021), Lee et al. (2023)
	CHMP4C (SNF7C)	CD63, CD81, MHC‐II, HSC70	Ju et al. (2021), Kenific et al. (2021)
	CHMP4B (SNF7B)	TSG101, RAB5, HRS	Stefanon et al. (2023)
	Alix	CD63, CD81, MHC‐II, HSC70, syndecan‐1, TSG101, RAB5, HRS	Ju et al. (2021), Kenific et al. (2021)
	VPS4	CD63, CD81, MHC‐II, HSC70, syndecan‐1	Kholodenko et al. (2022), Miura et al. (2022)
	Syntenin	CD63, HSP70	Cui et al. (2023)
	Syndecan	CD63, HSP70, Alix	Cui et al. (2023)
ESCRT‐independent	nSMase2	PLP, HRS, Tsg101	Betker et al. (2019), García‐Martínez et al. (2022), Komine‐Aizawa et al. (2020)
	PLD2	Syntenin, ALIX, CD63, SDC1CTF	García‐Martínez et al. (2022)
	DGKα	CD63, β‐Actin, Fasl	Komine‐Aizawa et al. (2020)
	CD9	β‐Catenin, Flotillin‐1	Komine‐Aizawa et al. (2020)
	CD82	β‐Catenin	Betker et al. (2019)
	CD63	HSC70, Calnexin, CD81	Cui et al. (2023), Han et al. (2024), Li et al. (2024)
	RAB31	Flotillin‐1, Flotillin‐2, CD9, CD81, CD63, Tsg101, Alix	Li et al. (2024)
Exosome release	RAB11	Transferrin receptor, Lyn, HSC70, Evi	Han et al. (2024)
	RAB27a/b	CD63, Tsg101, Hsc70, Hsp70, VLA‐4, Hsp90, Alix	Ju et al. (2021), Lee et al. (2023)
	RAB35	CD63, Tsg101	Lee et al. (2023)
	RalA, RalB	ALIX, CD63, HSC70, TSG101	Lee et al. (2023)
	VAMP7	Acetylcholinesterase activity	Han et al. (2024)
	YKT6	Tsg101	Han et al. (2024)
	Tetherin	CD63, ALIX, TSG101	Ju et al. (2021), Lee et al. (2023)

## 5 COMPONENTS OF EXOSOMES

**FIGURE 3:**
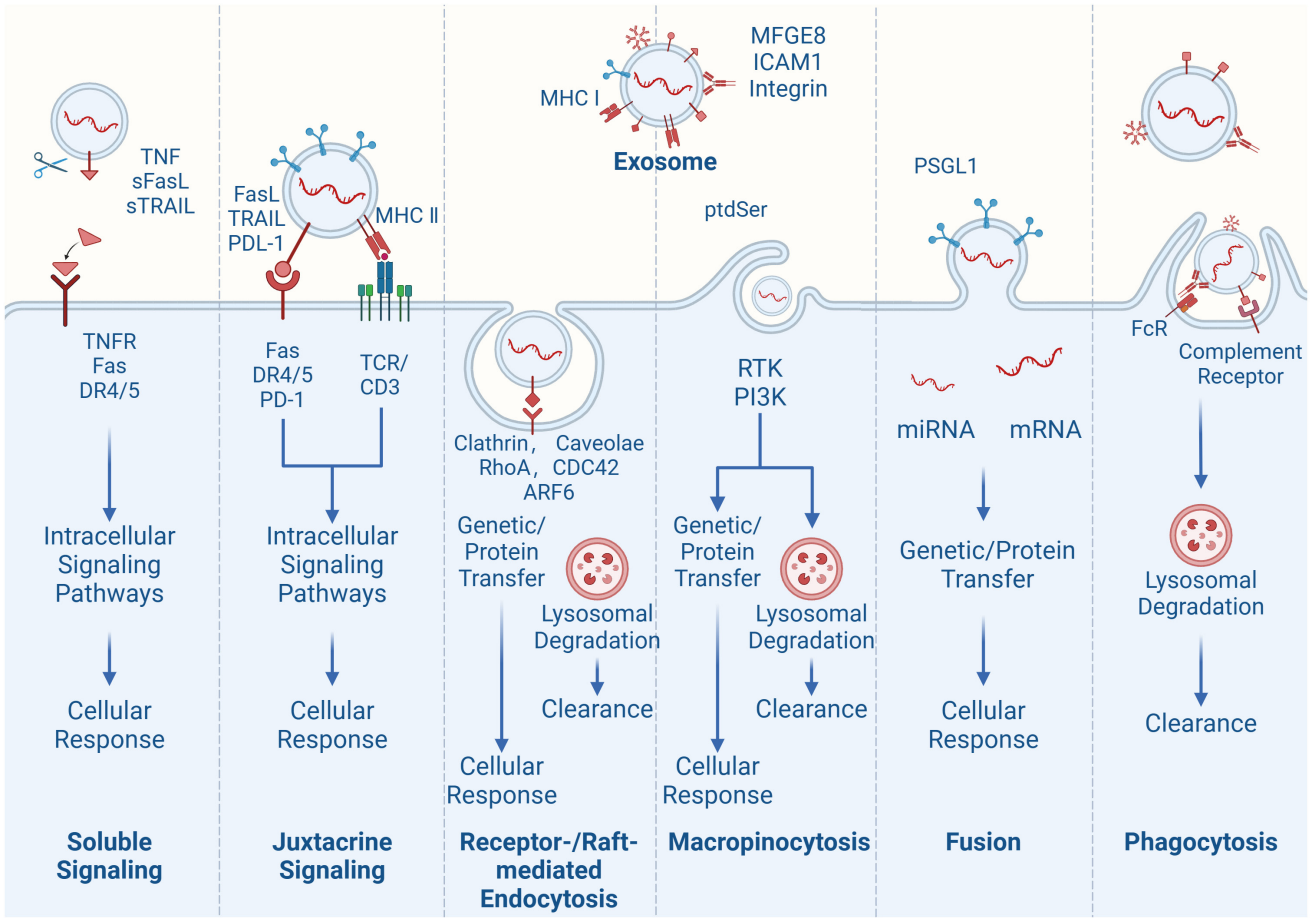
Influence of extracellular vesicles on receptor cell behavior via fusion, endocytosis, and signaling molecule release.

MDEs are characterized by a phospholipid bilayer that is similar to that of cells (Cui et al., 2023; Lu et al., 2024; Peng et al., 2023). This bilayer membrane is composed of sphingolipids, phosphatidylserine, cholesterol, and ceramides, which function to protect the contents of the vesicles from degradation and maintain their biological activity during long‐distance transport (Ahmed et al., 2022; Lu et al., 2024; Martínez‐Santillán & González‐Valdez, 2023). The bioactive components of exosomes are diverse and include proteins, nucleic acids (such as mRNA, miRNA, lncRNA, circRNA, and DNA), and lipids (Figure 2) (Ahmed et al., 2022; Cui et al., 2023; Lönnerdal, 2019). Moreover, MDEs are able to exert their influence on receptor cells via diverse signal transduction pathways, thereby engendering a highly intricate process (Figure 3) (Cui et al., 2023; Han et al., 2024; Lee et al., 2023). Notably, the bioactive components conveyed by different cells in varying states are not uniform (Ahmed et al., 2022; Cui et al., 2023; Zeng, Chen, et al., 2021). For example, bovine milk exosomes share common components with other exosomes, including Alix, Flillin1, the four transmembrane protein family (CD9, CD63, and CD81), integrins, and cell adhesion molecules (Del Pozo‐Acebo et al., 2021; García‐Martínez et al., 2022; Li et al., 2024). In addition, exosomes contain several proteins that are involved in their formation (Cui et al., 2023; García‐Martínez et al., 2022). The most common are testalin, Rab GTPase, and Tsg101, which control membrane fusion, interact with cytoskeletal proteins, and participate in endocytosis, respectively (Cui et al., 2023; García‐Martínez et al., 2022; Lu et al., 2024). Cow milk exosomes also express cytoskeletal proteins, such as actin, microtubulin, silk fibroin, heat shock proteins, and molecules involved in signal transduction (Betker et al., 2019; García‐Martínez et al., 2022; Komine‐Aizawa et al., 2020). Butyrate, lactase, and xanthine dehydrogenase are specific biomarkers of bovine milk extracellular vesicles (García‐Martínez et al., 2022). Furthermore, bovine milk exosomes have unique advantages over exosomes from other sources (Cui et al., 2023; García‐Martínez et al., 2022; Lu et al., 2024). Because of their origin in milk, the resistant glycoproteins (XDH, BTN, and MUC1) and surface proteins (FLOT1, ICAM1, ALIX, and EpCAM) of bovine milk exosomes render them resistant to pepsin and have good stability (Cui et al., 2023; García‐Martínez et al., 2022; Zeng, Chen, et al., 2021). Cow milk exosomes have been proven to withstand harsh environments such as gastric acid, low pHs, and high temperatures and can be used as delivery systems for oral drugs (Betker et al., 2019; Del Pozo‐Acebo et al., 2021). In addition, bovine milk exosomes have good transmembrane transport abilities, which helps them overcome the intestinal epithelial cell barrier and vascular endothelial barrier and allow them to enter blood circulation (Cui et al., 2023; Han et al., 2024; Li et al., 2024). Research shows that MDEs contain a high amount of stable ncRNAs that can be transferred to offspring and other consumers (Cui et al., 2023; Han et al., 2024; Li et al., 2024). These ncRNAs are believed to be involved in various biological processes and may have an epigenetic regulatory function in recipients (Cui et al., 2023; Zeng, Chen, et al., 2021). Zhang et al.'s research found six highly abundant miRNAs in human, cow, pig, panda, and sheep milk, including miR‐148a, let‐7a, let‐7b, let‐7f, miR‐30a, and miR‐30d (Zeng, Chen, et al., 2021). The above results indicate that MDE has a common molecule involved in interspecies information transmission and a molecular basis for regulating immune and nutritional metabolism functions (Komine‐Aizawa et al., 2020; Li et al., 2024; Stefanon et al., 2023). However, it should be noted that there may be differences in the composition of miRNAs in MDE, which may be related to factors such as nutrient concentration, variety, lactation period, sample processing, and sequencing analysis (Zeng, Chen, et al., 2021).

## 6 EXTRACTION OF EXOSOMES

**FIGURE 4:**
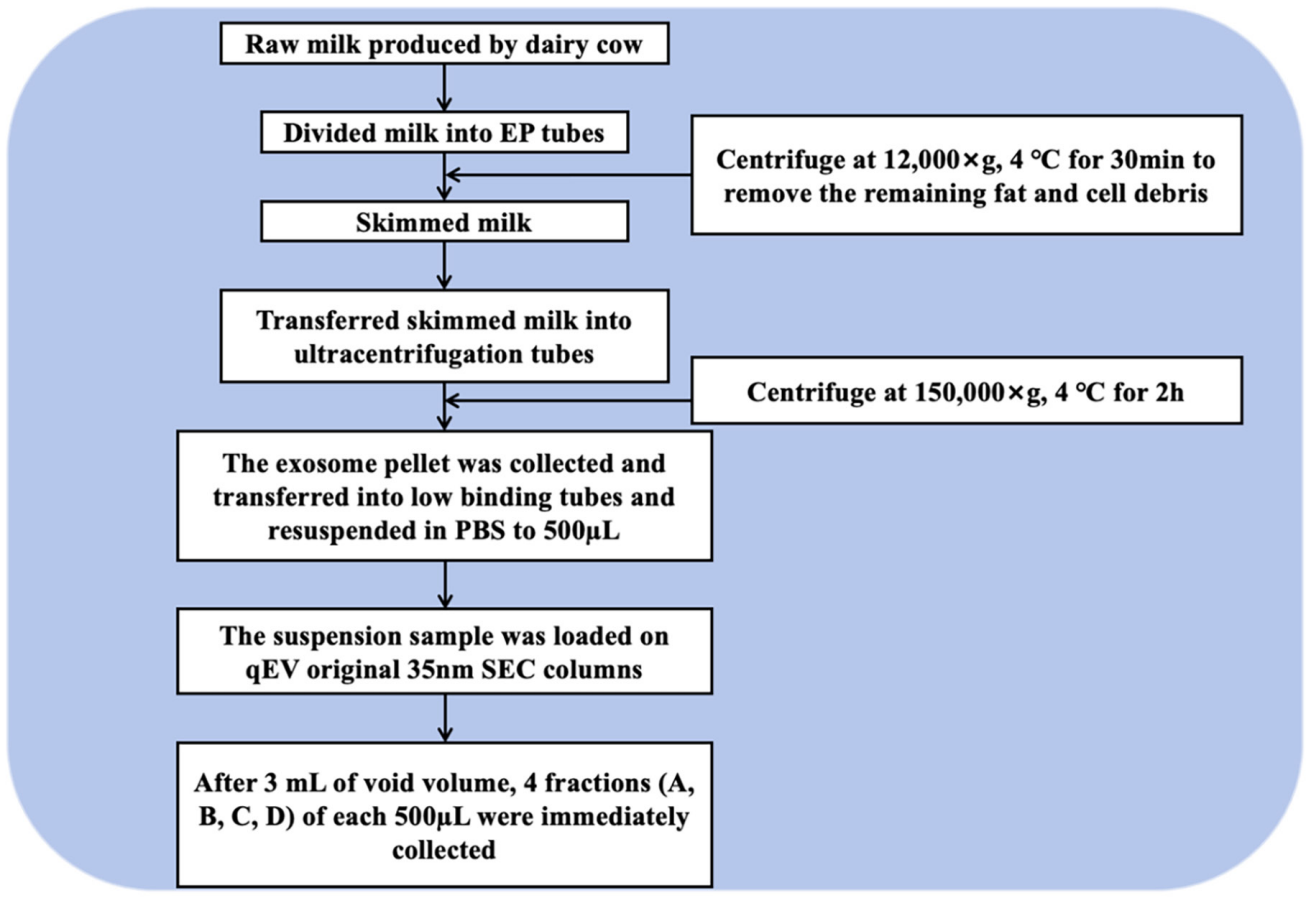
Isolation method for exosomes from bovine milk.

For MDEs‐related research, a rapid, simple, high‐purity, and high‐recovery isolation method is the primary prerequisite for the large‐scale application of exosomes in medical practice (Alzhrani et al., 2021; Qazi et al., 2023; Wang et al., 2023). Ultracentrifugation is the most common method for isolating exosomes from raw milk (Ferreira et al., 2021; Zeng, Chen, et al., 2021). Fat globules, dead cells, and bulky apoptotic debris are removed by centrifuging at 2000 × g, followed by precipitation of exosomes at 100,000–150,000 × g. The exosome pellets are then separated into four fractions using a size exclusion chromatography (SEC) column for further analysis. SEC is used to extract milk exosomes based on their size, often in combination with ultracentrifugation (Cui et al., 2023; Lu et al., 2024; Zeng, Chen, et al., 2021). Previous studies have also used density gradient centrifugation (DGC) in conjunction with ultracentrifugation and SEC to extract milk exosomes (Figure 4) (Zeng, Chen, et al., 2021). In a previous study, bovine milk‐derived exosomes were successfully separated using ultracentrifugation and SEC (Vaswani et al., 2017; Zeng, Chen, et al., 2021). The ExoQuick precipitation kit is more suitable for the separation of bovine milk exosomes compared to ultracentrifugation (Yamauchi et al., 2019; Zeng, Chen, et al., 2021). However, there is no unified standard for the separation and purification of bovine milk exosomes, and different milk sources can lead to differences in bovine milk exosomes (Vaswani et al., 2019; Yamauchi et al., 2019). In the course of extensive scientific practice, researchers have come to realize that different exosome preparation strategies can be chosen depending on the sample requirements for downstream studies, as well as the size and quantity of the starting sample (Yamauchi et al., 2019; Zeng, Chen, et al., 2021). Currently, isolating exosomes from milk using multiple methods may be better than using just one. In the DGC method, samples are added to a gradient medium and centrifuged to separate exosomes (Vaswani et al., 2019; Yamauchi et al., 2019; Zeng, Chen, et al., 2021). Furthermore, sucrose gradient centrifugation has been shown to effectively prevent the coprecipitation of nucleosomal fragments, apoptotic bodies, or protein aggregates (Livshits et al., 2015), resulting in improved separation efficiency compared to conventional methods and ultimately yielding exosomes of high purity (Witwer et al., 2013). However, additional research is necessary to establish and advance a standardized production platform for exosomes in milk in order to enhance their utilization, achieve higher concentrations and purities, and obtain a more comprehensive profile of exosomes derived from milk.

## 7 THE ROLE OF MDEs IN NUTRITIONAL REGULATION

The bioactive components of MDEs play important roles in intestinal health and nutritional regulation. Research has shown that MDEs can protect the intestines from harmful substances such as deoxynivalene (DON) (Xie et al., 2020). DON is often present in grain feed and has toxic effects on the intestines (Li et al., 2023). However, the active ingredients in MDEs can reverse the inhibitory effects of DON on cell growth and apoptosis, reduce damage to intestinal epithelial cells and reduce their negative impact on body weight (Hou et al., 2024). MDEs also show potential in the treatment of diseases caused by malnutrition (Melnik & Schmitz, 2019; Redwan & Uversky, 2022; Zempleni, 2017). The study found that treatment with MDEs improved intestinal permeability, intestinal structure, and cell proliferation, thus reducing pathological changes caused by malnutrition in mice fed a low‐protein diet (Li et al., 2024; Stremmel et al., 2020). MDEs also exert positive effects on intestinal inflammation and stress damage (Cui et al., 2023; Gao et al., 2021; Han et al., 2024; Stremmel et al., 2020). Research has shown that under hypoxic conditions, specific miRNAs carried in MDEs can reduce the expression of hypoxia‐inducible factor 1a (HIF‐1a) and its downstream vascular endothelial growth factor in intestinal epithelial cells, thereby reducing the damage of hypoxia to the intestine and promoting the recovery of intestinal digestion and absorption function (Gao et al., 2021). Additionally, research has found that MDEs have a positive impact on the bioavailability and therapeutic efficacy of drugs (Ahmed et al., 2022; Alzhrani et al., 2021; Betker et al., 2019). For example, loading insulin into MDEs for oral administration can improve its absorption and stability in the intestine, thereby achieving improved and long‐lasting therapeutic effects (Wu et al., 2022). This indicates that MDEs can increase the bioavailability of drugs in the intestine and participate in the regulation of nutrients such as blood sugar (Betker et al., 2019; García‐Martínez et al., 2022; Wu et al., 2022).

However, some MDE components may have negative effects. For example, miR‐21 in MDEs can activate and accelerate fetal macrosomia, leading to excessive nutrient synthesis and rapid fetal growth (Jiang et al., 2014; Lönnerdal, 2019; Zhang et al., 2016). Additionally, some MDE components may promote the proliferation and differentiation of adipocytes, leading to fat accumulation and obesity (Cho et al., 2016; Lönnerdal, 2019; Shi et al., 2016). Breast milk exosomes containing high levels of transforming growth factor‐β2 (TGFβ2) induce changes in both benign and malignant breast epithelial cells, which is consistent with the development and progression of breast cancer and suggests a role for high TGFβ2‐expressing breast milk exosomes in influencing breast cancer risk (Hannafon et al., 2016; Lowry et al., 2015; Miura et al., 2022; Ramezani et al., 2023; Zeng, Wang, et al., 2021). Continuous intake of milk exosomes may pose a risk for chronic diseases, including obesity, type 2 diabetes mellitus, osteoporosis, Parkinson's disease, and common cancers, mainly because of miRNAs inside the exosomes, such as miR‐148a which suppresses adipogenesis, miR‐29b which belongs to the diabetogenic miR family, miR‐155 which can promote the initiation and progression of Parkinson's disease in humans, and miR‐21 which promotes tumor progression (Fasken et al., 2020; Melnik & Schmitz, 2019; Ramezani et al., 2023; Wang et al., 2017). In summary, the bioactive components of MDEs play important roles in intestinal health and nutritional regulation. They protect the intestine from harmful substances, improve the bioavailability of drugs, and have therapeutic potential in diseases such as malnutrition, inflammation, and stress damage (Redwan & Uversky, 2022; Wu et al., 2022; Zempleni, 2017).

## 8 THE ROLE OF MDEs IN REGULATING IMMUNE FUNCTION

In addition to their role in nutritional regulation, MDEs play an important role in immune regulation (Cui et al., 2023; Gao et al., 2021; Han et al., 2024). MDEs regulate immune function by modulating the number of immune cells (Komine‐Aizawa et al., 2020). Admyre et al. (2007) first discovered that human breast MDEs could promote the differentiation of regulatory T cells, suggesting a potential role for exosomes in immune cell regulation (Admyre et al., 2007). Exosomes in pig colostrum promote the proliferation of cytotoxic T cells and increase the proportion of these cells in the body (Miura et al., 2022). Zeng et al. discovered that pig MDEs can increase the secretion of secretory immunoglobulin A in the intestine and enhance the expression of polymeric immunoglobulin receptors in mice and piglets, thus participating in intestinal mucosa homeostasis and the formation of acquired immunity (Zeng, Wang, et al., 2021).

**FIGURE 5:**
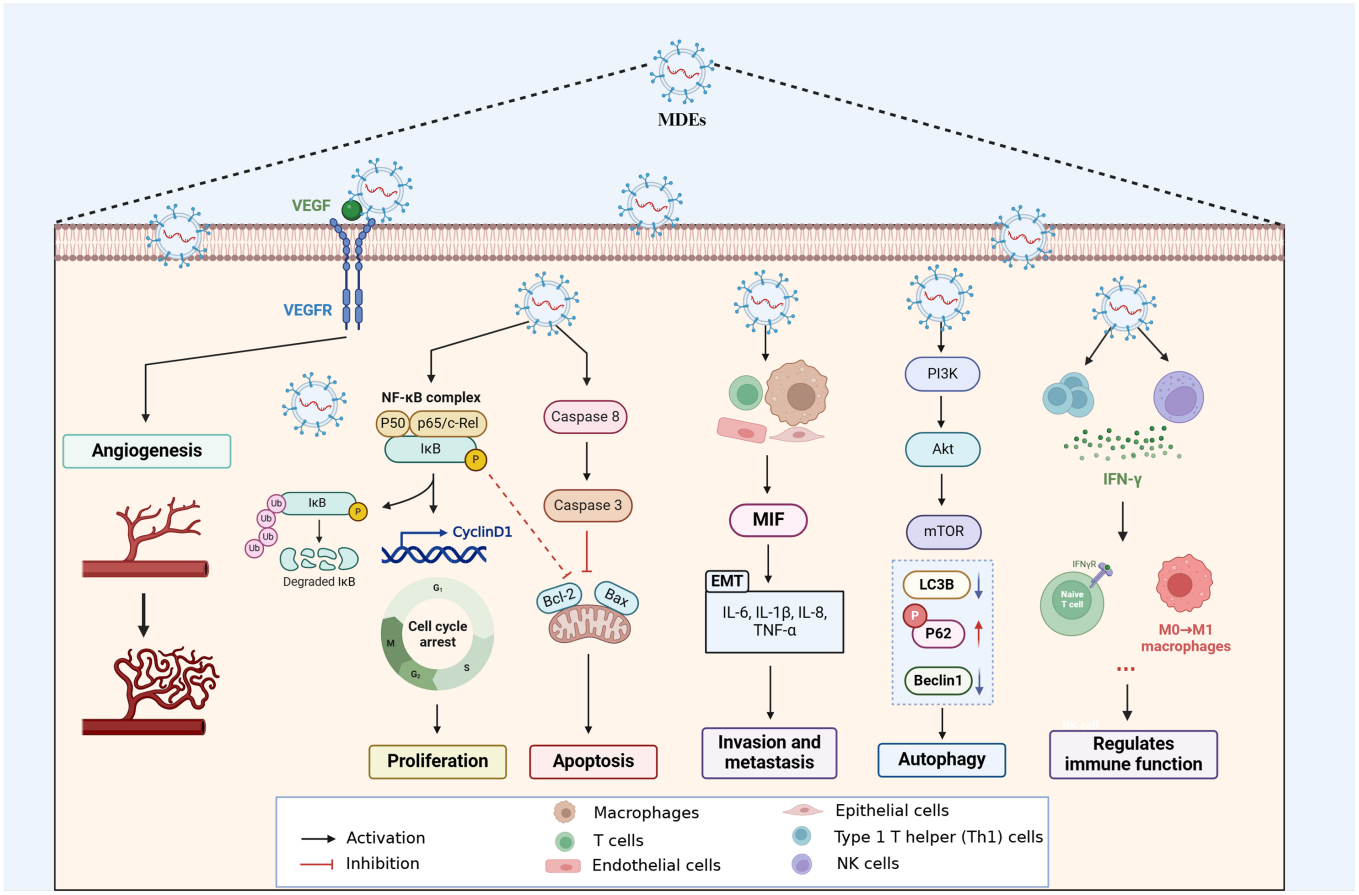
Model of immune regulatory mechanism of milk‐derived exosomes in the intestinal epithelium.

Numerous research reports have elucidated the regulatory mechanism of milk‐derived exosomes in intestinal diseases (Figure 5) (Cui et al., 2023; Gao et al., 2021; Han et al., 2024; Zeng, Chen, et al., 2021). An examination was conducted on the miRNA expression in human breast milk, revealing elevated levels of immune‐related miRNAs during the initial 6 months of lactation (Lönnerdal, 2019; Słyk‐Gulewska et al., 2023). These miRNAs were found to play a crucial role in regulating the development of the intestinal immune system in infants (Fasken et al., 2020; Miyake et al., 2020). Additionally, research has indicated that exosomes derived from breast milk are abundant in transforming growth factor β (TGF‐β), which plays a key role in the development of intestinal barrier function, the production of immunoglobulin A (IgA), and mucosal immunity in infancy (Pieters et al., 2015). Research conducted both in vitro and in vivo has shown that peptides abundant in milk‐derived exosomes have the potential to mitigate ileal damage by stimulating the proliferation and migration of intestinal cells, offering a promising preventive approach for necrotizing enterocolitis (NEC) (Cui et al., 2023; Han et al., 2024; Zeng, Wang, et al., 2021). Furthermore, studies have indicated that human breast milk‐derived exosomes can safeguard intestinal stem cells from oxidative stress‐induced damage through the activation of the Wnt/β‐catenin signaling pathway, thereby presenting a potential therapeutic strategy for the prevention and treatment of NEC (Dong et al., 2020). Studies have shown that human breast milk‐derived exosomes have been found to significantly enhance intestinal epithelial cell (IEC) proliferation, inhibit apoptosis, and reduce the severity and occurrence of NEC (Martin et al., 2018). Additionally, research has demonstrated that human breast milk‐derived exosomes protect IECs, promote cell viability by mitigating oxidative stress, and prevent the development of NEC and intestinal injury (Cui et al., 2023; Han et al., 2024; Zeng, Chen, et al., 2021). Human milk‐derived exosomal long noncoding RNAs (lncRNAs) and messenger RNAs (mRNAs) have been shown to prevent NEC by promoting intestinal tissue proliferation and development, reducing tissue necrosis and epithelial injury, and mitigating the severity of NEC through the activation of the JAK–STAT and adenosine monophosphate‐activated protein kinase (AMPK) signaling pathways (Yan et al., 2022). Additionally, human milk‐derived exosomal lipids have been found to alleviate the severity of NEC by activating the extracellular signal‐regulated protein kinase/mitogen‐activated protein kinase (ERK/MAPK) pathway, which rescues the apoptosis and migration inhibition of intestinal epithelial cells induced by lipopolysaccharide (LPS) (Chen et al., 2021). Previous research has shown that exosomes derived from human milk have been found to mitigate hypoxia and LPS‐induced NEC inflammation, mucosal damage, and mucus production (Gao et al., 2019). Additionally, it has been demonstrated that exosomal miR‐148a‐3p from human milk plays a role in preventing NEC by upregulating Sirtuin 1 and downregulating p53 and NF‐κB expression (Guo et al., 2022).

While exerting positive immunoregulatory effects, MDEs also negatively affect immune regulation, particularly in immune‐related diseases; MDEs are also associated with various allergic diseases (Engeroff & Vogel, 2022; Ma, Xia, Yuan, et al., 2024; Yu et al., 2017). Research has shown that the development of newborn allergen tolerance and termination of allergic reactions in the body are closely related to the demethylation of the human forkhead box P3 (FOXP3) protein, which is regulated by DNA methyltransferase 1 (DNMT1) and DNA methyltransferase 3 B (DNMT3B) (Ma, Xia, Gong, et al., 2024; Ma, Xia, Yuan, et al., 2024; Pan et al., 2010). MiRNA‐21 acts on its target Ras guanyl‐releasing protein 1 (RasGRP1) and indirectly reduces DNMT1 expression (Jiang et al., 2014; Zhang et al., 2023). In contrast, miRNA‐148a negatively regulates DNMT1 expression and increases the risk of allergic diseases such as allergic rhinitis, allergic asthma, and allergic dermatitis (Ahmed et al., 2022; Cañas et al., 2021; Pan et al., 2010). In summary, MDEs play significant roles in immune regulation. They participate in the development of immune functions and the modulation of immune cell numbers. They also contribute to intestinal mucosal homeostasis and acquired immunity.

## 9 CONCLUSIONS AND FUTURE PERSPECTIVES

MDEs have garnered significant attention in academic research in recent years as a natural bioactive substance, owing to their robust biocompatibility, stability, and safety. Furthermore, these vesicles possess the ability to be assimilated by the body, thereby sustaining biological activity and facilitating the transmission of biological information to target cells. This functionality allows MDEs to play a pivotal role in immune regulation in conditions such as inflammation, cancer, and cell proliferation. Nevertheless, ongoing studies on MDEs encounter challenges related to the separation and purification process, including variations in purity, yield, and cost. The need for enhanced methods to isolate and purify MDEs with high efficiency remains a critical area for advancement, impacting the precision and reliability of research in this field. Consequently, the development of novel techniques for separation and purification continues to be a primary objective in current research endeavors. In the investigation of the immune regulatory function of MDEs, current research predominantly centers on the characterization of the MDEs population, with limited exploration of their individual functional constituents, particularly miRNAs. Given the greater sequence homology of miRNAs in comparison to other biomolecules such as lncRNA, circRNA, proteins, and lipids, delving into the study of these alternative functional components within MDEs has the potential to yield significant advancements in the field of MDEs research. Of particular concern is the potential transfer of viral RNA or DNA. Therefore, caution should be exercised when employing extracellular vesicles as adjunctive immunomodulatory treatments, due to the associated risks.

## AUTHOR CONTRIBUTIONS

**Hui Yang:** Conceptualization (lead); data curation (equal); formal analysis (equal); investigation (equal); methodology (equal). **Tana Wuren:** Conceptualization (equal); data curation (equal); formal analysis (equal); funding acquisition (equal); investigation (equal); project administration (equal). **Bin‐tao Zhai:** Conceptualization (equal); data curation (equal); formal analysis (equal). **Yang Liu:** Data curation (equal); formal analysis (equal); methodology (equal). **Demtu Er:** Data curation (equal); formal analysis (equal); investigation (equal).

## FUNDING INFORMATION

This work was supported by the Sichuan Science and Technology Program (Grant No. 2023YFQ0068).

## CONFLICT OF INTEREST STATEMENT

The authors declare that the research was conducted in the absence of any commercial or financial relationships that could be construed as a potential conflict of interest.

## ETHICS STATEMENT

The animal study was approved by Qinghai University Animal Ethics Committee. This study was conducted in accordance with the local legislation and institutional requirements.

## Data Availability

The raw data supporting the conclusions of this article will be made available by the authors, without undue reservation.
